# In Vitro Antimycotic Activity and Structural Damage against Canine *Malassezia pachydermatis* Strains Caused by Mexican Stingless Bee Propolis

**DOI:** 10.3390/vetsci11030106

**Published:** 2024-02-28

**Authors:** Diana Berenice Fuentes Esquivel, Betsabé Rodríguez Pérez, Nelly Tovar Betancourt, Carlos Gerardo García Tovar, José Guillermo Penieres Carrillo, Florentina Hernández Galindo, Javier Pérez Flores, Tonatiuh Alejandro Cruz Sánchez

**Affiliations:** 1 Laboratorio de Servicio de Análisis de Propóleos (LASAP®), Facultad de Estudios Superiores Cuautitlán, Campo 4, National Autonomous University of México, Km 2.5 Carretera Cuautitlán-Teoloyucan, San Sebastián Xhala, Cuautitlán Izcalli 54714, State of México, Mexico; db_ciani@hotmail.com (D.B.F.E.); betsarguez79@gmail.com (B.R.P.); nelly.tovar@cuautitlan.unam.mx (N.T.B.); 2 Laboratorio de Morfología Veterinaria y Biología Celular, Facultad de Estudios Superiores Cuautitlán, Campo 4, National Autonomous University of México, Km 2.5 Carretera Cuautitlán-Teoloyucan, San Sebastián Xhala, Cuautitlán Izcalli 54714, State of México, Mexico; cgarciatov@yahoo.com.mx; 3 Laboratorio de Química Orgánica, Facultad de Estudios Superiores Cuautitlán, Campo 1, National Autonomous University of México, Av. 1o de Mayo s/n, Santa María las Torres, Cuautitlán Izcalli 54714, State of México, Mexico; penieres@unam.mx; 4 Colegio de la Frontera Sur (ECOSUR) San Cristóbal de las Casas, Panamerican Highway Periférico Sur s/n, Barrio María Auxiliadora, San Cristóbal de las Casas 29290, Chiapas, Mexico; florentina.hega@gmail.com; 5 Laboratorio de Espectrometría de Masas, Instituto de Química, National Autonomous University of México, Circuito exterior s/n Circuito de la Investigación Científica, Ciudad Universitaria, México City 04510, Mexico; japeflo10@hotmail.com

**Keywords:** Mexican stingless bee, propolis, antimicotic activity, structural damage, *Malassezia pachydermatis*

## Abstract

**Simple Summary:**

Surprisingly, there is little research on the antimicrobial activity of stingless bee propolis on disease-causing microorganisms in animals. The present work demonstrates the effect of propolis from two native Mexican bees, *Scaptotrigona mexicana* and *Tetragonisca angustula*, on the yeast *Malassezia pachydermatis*, the main agent of canine otitis externa, and its damage to the cellular structure. The chemical analysis showed that the most abundant components are some sesquiterpenes. The antifungal activity of the propolis was evaluated using both strains isolated from clinical cases and a reference strain. Both types of propolis inhibited all Malassezia pachidermatis strains. Cell damage was assessed by fluorescence microscopy with calcofluor white, which specifically stains the fungal cell wall, and propidium iodide, which has the ability to stain the interior of the cell, only if the cell wall or membrane has been damaged. The propidium iodide staining of the yeasts treated with both types of propolis revealed the penetration of this marker, which suggests the destruction of the cell wall and plasma membrane of the fungi. These results suggest that these types of propolis could be used as alternative treatments for canine external otitis. This seems to be the first scientific report that has demonstrated structural damage in *Malassezia pachydermatis* by Mexican stingless bee propolis.

**Abstract:**

This work describes the antimycotic activity of propolis from the stingless bees *Scaptotrigona mexicana* and *Tetragonisca angustula*, collected from two Mexican regions (Veracruz and Chiapas, respectively), against three clinical isolates and the reference strain ATCC 14522 of *Malassezia pachydermatis*, the causative agent of canine otitis. The chemical components of the ethanolic extracts of propolis were determined by gas chromatography coupled with mass spectrometry (GC-MS), and sesquiterpenes were the predominant compounds. The antimycotic activity was evaluated by plate microdilution. The induced changes in the yeasts were evaluated by fluorescence microscopy and staining with calcofluor white and propidium iodide. The minimum inhibitory concentration (MIC) was 7.11 mg/mL, and the minimum fungicidal concentration was 21.33 mg/mL for both extracts. The EPPs of *Scaptotrigona mexicana* and *Tetragonisca angustula* caused substantial damage to yeast morphology, where the propidium iodide staining of the yeasts treated with both EEPs revealed the penetration of this marker, which indicates the destruction of the cell wall and plasma membrane of the fungi. This result suggests that these types of propolis could be used as alternative treatments for canine external otitis. To the best of our knowledge, this seems to be the first scientific report that has demonstrated structural damage in *Malassezia pachydermatis* by Mexican stingless bee propolis.

## 1. Introduction

Propolis is a natural resinous substance produced by bees. Propolis is derived from substances collected by bees from vegetation and shows antifungal, antibacterial, antiviral, and antiparasitic activities. Propolis shows variation in its biological activity depending on its geographical origin [1,2,3]. In Latin America, there is a great variety of ecosystems with diverse vegetation from which native bees extract propolis, which in turn results in exceptional medicinal richness. For this reason, chemotaxonomic studies on native bees in Mexico are scarce, despite the fact that there are 46 species [4]. 

In general, identifying the origin of the material with which bees produce propolis should be carried out, and the behaviour of each bee species in a region should be observed [5,6,7]. Since ancient times, products elaborated by the stingless bees *Scaptotrigona mexicana* and *Tetragonisca angustula* have been used in Central America; however, there is little scientific evidence demonstrating the medicinal efficacy of these products. In comparison, the propolis produced by *Apis mellifera* has been extensively studied, and its fungicidal effects have been reported [8,9,10].

Moreover, the application of propolis can prove beneficial in veterinary medicine, for instance, with dogs. Propolis from *Apis mellifera* has been used as a prophylactic agent against gastrointestinal and respiratory diseases and mycoses, as well as a wound-healing agent, and its therapeutic use has spread to many areas [11,12], such as the treatment of canine external otitis. This condition can be defined as the inflammation of the external auditory canal and represents between 5% and 20% of consultations. The main causative agent of canine otitis externa is the yeast *Malassezia pachydermatis*, which is part of the normal microbiota of the external auditory canal in dogs [13,14,15,16]. 

Propolis can be an alternative to conventional antifungals for the treatment of canine otitis in patients with a high incidence of relapse because of its antifungal, antiinflammatory, and wound-healing properties. However, only the propolis obtained from *Apis mellifera* has been evaluated so far [17]. The antifungal activities of the propolis of other bee species and that of *Melipona beecheii* have been reported in Mexico; however, only their antifungal activities against *Candida albicans* were evaluated. It is important to mention that this activity is attributed to compounds such as sesquiterpenes and flavonones [2,3].

In addition to the inhibitory effect of a compound, possible damage to the microbial cell structure should be evaluated. As we demonstrated in a previous study by scanning electron microscopy, *Apis mellifera* propolis produced the formation of pores and promoted the destruction of the cell structure of *Malassezia pachydermatis* [18]. In this work, we will use fluorescence microscopy using calcofluor white and propidium iodide stains to detect cell damage, as other authors have done with fungi [19].

Research on the antimicrobial activity of stingless bee propolis on disease causing microorganisms in animals is scarce; therefore, studies on the antifungal potential of propolis in this type of bee may be of veterinary interest. Therefore, the aim of this study is to provide data regarding the antimycotic properties of propolis from native bees.

We expect that this study will provide scientific evidence that supports the use of propolis as an alternative treatment for canine otitis.

## 2. Materials and Methods

### 2.1. Ethanolic Extract of Propolis (EEP)

Propolis samples were obtained from *Scaptotrigona mexicana* and *Tetragonisca angustula* stingless bees. The *S. mexicana* sample was from Yecuatla, Veracruz, Mexico, located at 19°51 N and 96°46 W, at an altitude of 432 m.a.s.l. The *T. angustula* sample was from Chalchihuitan, Chiapas, located at 16°57 N and 92°37 W, at an altitude of 1461 m.a.s.l. The collected material was evaluated for its physical properties according to Mexican regulations regarding colour, odour, taste and consistency [20]. Propolis from *S. mexicana* (30 g) and *T. angustula* (12 g) was weighted, and any present impurities were eliminated. Thereafter, 100 mL of 70% ethanol was added to each sample, and the obtained mixture was subjected to ultrasonic extraction (Branson, CPX1800H, Danbury, CT, USA). Each sample was then vacuum-filtered, and the obtained filtrates were concentrated using a rotary evaporator (Science MED, SM100-PRO, Helsinki, Finland) and dried with a vacuum pump. Then, both dried extracts were placed in light-resistant containers and kept at 4 °C until use [21].

### 2.2. Gas Chromatography–Mass Spectrometry (GC-MS)

A chromatographic analysis of ethanolic extracts was performed using a gas chromatograph (6850) coupled to a mass spectrometer (7890 model, JEOL MC-GC-Mate II, Tokyo, Japan). A HP-5MS (30 m × 0.32 mm) capillary column and a film thickness of 0.25 µm were used. Helium gas was used as the carrier gas. The elected injection method was split mode with an injection volume of 1 µL. The separation conditions were as follows: 70 °C at the beginning for two minutes, followed by two ramp increments. The first one was an increase of 20 °C per minute until a temperature of 230 °C was reached; the second one was an increase of 8 °C per minute until a temperature of 290 °C was reached, keeping this temperature for a period of 5 min. The total analysis time was 21.25 min. The detected mass range was 35 m/z to 750 m/z, and each sample was subjected to electron impact ionisation at 70 eV, with the ionisation source reaching a temperature of 230 °C. Compound identification was carried out by comparison with the library database from the equipment [22].

### 2.3. Evaluation of Antimycotic Activity

#### 2.3.1. Inoculum Preparation

Four *Malassezia pachydermatis* strains were used: ATCC 14522 and three clinical isolates from three German Shepherd dogs, two females and a male of three years of age, which presented symptoms of otitis externa with an accumulation of abundant foul-smelling ceruminous secretion. The patients were sampled on the premise that they had not received any treatment for otitis externa. The sample was obtained with a sterile swab and placed in a tube with Sabouraud Dextrose broth as a transport medium. A first seeding was carried out on modified Dixon Agar and incubated at 33 °C for 72 h, after which a reseeding was carried out on Sabouraud Dextrose Agar (SDA) and incubated at 33 °C for 72 h.

All strains were identified by biochemical testing [23]. Microorganisms were provided by the Laboratorio de Servicio de Análisis de Propóleos (LASAP^®^) of Facultad de Estudios Superiores Cuautitlán, Universidad Nacional Autónoma de México. To activate the *M. pachydermatis* strains, each type of yeast was seeded in modified Dixon agar (mDA). Each type of yeast was seeded in a different Petri dish and incubated for 72 h at 33 °C. Then, samples were reseeded in other mDA-containing plates and incubated for 48 h at 33 °C to rule out strain contamination [23,24]. A roast of the colonies sown with yeast was taken on Sabouraud Dextrose Agar (SDA) supplemented with 2% glucose (Bioxon, Monterrey, Mexico). It was incubated at 33 °C for 48 h. The inoculum density was adjusted according to the 0.5 tube of the MacFarland Nephelometer (1.5 × 10^6^ cells/mL), comparing turbidity in a spectrophotometer at 625 nm with an absorbance between 0.08 and 0.10. From this inoculum (1.5 × 10^6^ cells/mL), 100 μL was taken and placed in a tube with 9.9 mL of Dextrose Sabouraud broth to obtain a concentration of 1.5 × 10^3^ CFU/mL [10].

#### 2.3.2. Determination of Minimum Inhibitory Concentration and Minimum Fungicidal Concentration

The procedure of the microtechnique of dilution in broth was carried out according to the M27-A3 microdilution protocol for *Candida* spp., with adaptations for *Malassezia pachydermatis* [25,26]. Microdilution in broth was performed by determining the minimum inhibitory concentration (MIC) and the minimum fungicidal concentration (MFC). To this end, serial double dilutions of each EEP were performed to evaluate concentrations from 0.0001 to 21.33 mg/mL. Then, 50 μL of the inoculum of 10^3^ CFU/mL was added to each well. The positive control was broth with microorganisms, and as a negative control, only broth was used, and the plate was subsequently incubated at 33 °C for 48 h. To detect the respiratory activity of *Malassezia pachydermtis*, a 0.08% solution of 2,3,5-Triphenyltetrazolium chloride for microbiology (TTC) (MERK, Darmstadt, Germany) was used, which generates a red pigment (formazan) in the presence of microorganisms. This procedure was performed as follows: 50 μL of TCC was added to each well, inoculated, mixed using a plate stirrer, and incubated at 33 °C for 30 min. After this time, the formation of an insoluble red precipitate was observed, representing the MIC. The CMF was determined in the well where no color developed, indicating that there was no yeast growth. To confirm the results, it was determined whether the effect was fungicidal by taking a sample of the crop with a loop and seeding it in an SDA plate that was kept in incubation at 33 °C for 48 h. The growth on the plate was considered to be indicative of a fungistatic effect, while its absence corresponds to a fungicidal effect [10].

### 2.4. Structural Damage

To evaluate the structural changes induced by the EEP on *Malazassia pachidermatis*, fluorescence microscopy was used, and the reference strain and one clinical strain were employed. A concentration of 21.3 mg/mL of each EEP of *Scaptotrigona mexicana* and *Tetragonisca angustula* was added to each strain. The concentration used was the minimum fungicide concentration obtained in the antifungal evaluation. Incubation was carried out at 33 °C for 48 h. When the incubation ended, the yeast was stained with calcofluor white (M2R 1 g/L, (Sigma Aldrich, St. Louis, MO, USA) and propidium iodide (2.4 mmol/L, (Sigma Aldrich, St. Louis, MO, USA))**,** and as a negative control, a culture without EEP was used. Calcofluor white staining stains the yeast wall blue and allows its integrity to be evaluated; propidium iodide binds to DNA, stains it red, and only penetrates the cells if there is damage to the cell wall, so cells damaged with EEP do not stain with calcofluor white due to damage to the cell wall, and they are stained with propidium iodide. Preparations were viewed on a microscopy Zeiss Axioscop 40, coupled to an Evolution VF Cooled Color camera from Media Cibernetics (Silver Spring, MD, USA). All experiments were performed in triplicate [19,27].

## 3. Results

### 3.1. Gas Chromatography–Mass Spectrometry (GC-MS)

**Figure 1:**
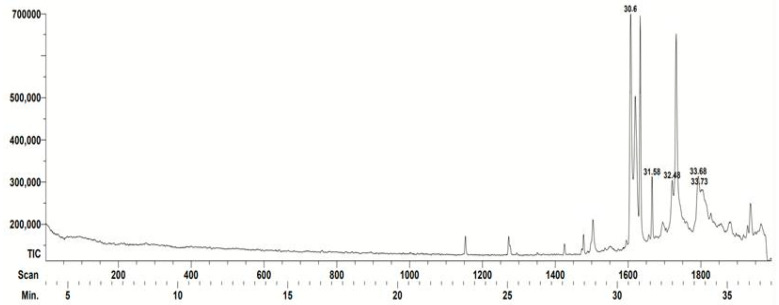
Gas chromatogram corresponding to the *Scaptotrigona mexicana* propolis. The retention time of each compound identified by the database is indicated (minutes): 30.6: 1,4-Methanocycloocta[d]piridazine, 1,4, 4a,5,6,9,10,10a-octahydro-11,11-dimethyl-,(1-alpha.,4-alpha,4a-alfa,10a-alfa)-; 31.58: Farnesol isomer a; 32.48: Ethanone, 1-(1,3a,4,5,6,7-hexahydro-4-hydroxy-3,8-dimethyl-5-azulenyl)-; 33.68: 2*H*-1-Benzoxacyclohexadecin-16(18a*H*)-one,3,4,5,6,7,8,9,10,11,12,13,14-dodecahydro-18,18a-dihidroxy-2-methyl; and 33.73: Furan-2,5-dicarbaldehyde. The characteristics of the compounds can be found in Table 1. Spikes that are not numbered were not identified by the team’s database.

The chemical composition of *S. mexicana* propolis extract is shown in Table 1. The database identified five compounds, two sesquiterpenes with antimicrobial activity, a heterocyclic compound called pyridazine with antioxidant properties, a macrocycle, and a compound of the furan class which has no information of any biological activity so far. The database detected other peaks but failed to identify them (Figure 1). 

**Figure 2:**
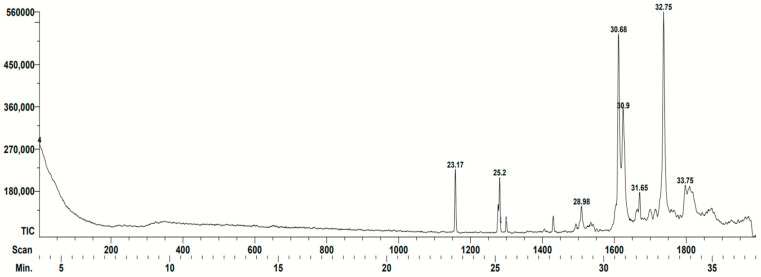
Gas chromatogram corresponding to the *Tetragonisca angustula* propolis. The retention time of each compound identified by the database is indicated (minutes): 23.17: Hexadecanoic acid ethyl ester; 25.20: 9-Octadecenoic acid, ethyl ester; 28.98: 1-(1,1-dimethylethoxy)-4-methylbenzene; 30.68: Solavetivone; 30.90: Benzene, 1-(1,1-dimethylpropoxy)-4-methyl; 31.65: 2-Methyl-3-(3-methyl-but-2-enyl)-2-(4-methyl-pent-3-enyl)-oxethane; and 32.75: (1S,6R,9S)-5,5,9,10-etramethyltricy-cle[7.3.0.0(1,6)]dodec-10(11)-ene. The characteristics of the compounds can be found in Table 2. Spikes that are not numbered were not identified by the team database.

In the case of the *T. angustula* sample, only five compounds with biological activity were identified (Table 2). Terpenes with antifungal activity can be appreciated. It is also a compound with antibacterial activity (1,3-Benzenediol, 5-hexyl) as well as one with nematicide capacity (Hexadecanoic acid ethyl ester) The main peaks identified are shown in Figure 2.

### 3.2. Evaluation of the Antimycotic Activity

All the tested *Malassezia pachydermatis* strains were susceptible to the propolis extracts. The minimum inhibitory concentration (MIC) was 7.11 mg/mL, and the minimum fungicidal concentration (MFC) was 21.33 mg/mL (Table 3).

### 3.3. Structural Damage

The structural damage to the yeasts was determined by using different stains: calcofluor white and propidium iodide. The structural damage of yeasts was determined by using different stains: calcofluor white and propidium iodide. Yeasts stained blue (calcofluor white) indicate integrity of the cell wall (control), those stained red (propidium iodide) indicate damage to the cell wall, since the damage wall allows the entry of propidium iodide (red) (EEP exposed). Propidium iodide penetration indicates damage to the yeast and with calcofluor-white stain, only morphology deformation was observed. In the untreated control cultures, the yeasts were clearly stained blue with calcofluor-white, but not with propidium iodide, which indicates the integrity of the yeast cell.

**Figure 3:**
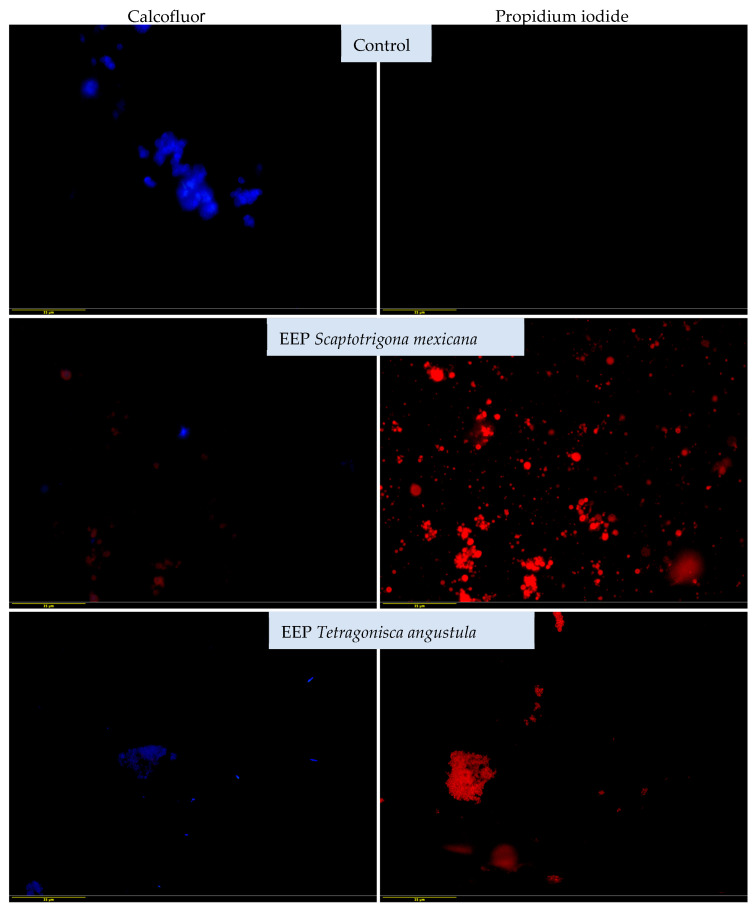
Effects of EEP of *Scaptotrigona mexicana* and *Tetragonisca angustula* on reference strain *Malassezia pachydermatis* ATCC 14522 were obtained by fluorescence microscopy and dyeing with calcofluor white and propidium iodide. Cultures were exposed to EEP at a concentration of 21.3 mg/mL for 48 h at 28 °C. Yeast samples stained in blue (calcofluor white) indicate the integrity of the cell wall (control), while those stained in red (propidium iodide) indicate damage to the cell wall. They do not stain in blue due to damage to the cell wall, which allows the entry of propidium iodide (red) (EEP exposed). Propidium iodide penetration was observed, indicating damage to the yeast. With the calcofluor white stain, only morphology deformation was observed (40× magnification).

Figure 3 illustrates the effects of both propolis extracts on the reference strain of *M. pachydermatis* (ATCC 14522). The yeast samples treated with the EEP of *S. mexicana* did not show staining with calcofluor white but showed staining with propidium iodide in the form of red colouration, with the most severe damage being that produced by the EEP of *S. mexicana*.

**Figure 4:**
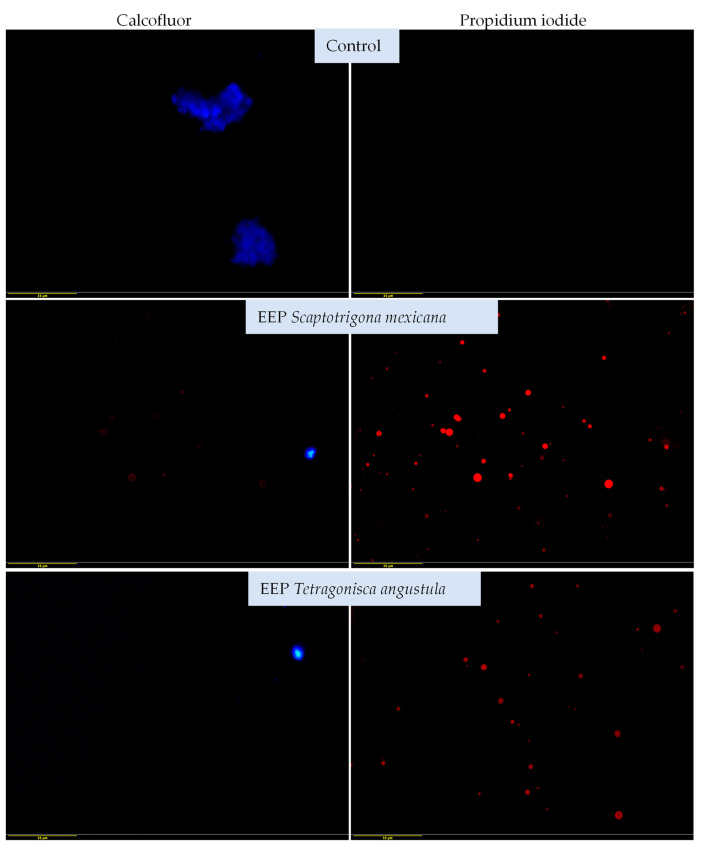
Effects of the EEPs of *Scaptotrigona mexicana* and *Tetragonisca angustula* on the clinical strain of *Malassezia pachydermatis*, as determined by fluorescence microscopy and dyeing with calcofluor-white and propidium iodide. Cultures were exposed to 21.3 mg/mL of EEP for 48 h at 28 °C. Alteration of morphology is observed with the calcofluor white stain. As in Figure 3, yeast samples stained in blue (calcofluor white) indicate the integrity of the cell wall (control), while those stained in red (propidium iodide) indicate damage to the cell wall. They do not stain in blue due to damage to the cell wall, which allows the entry of propidium iodide (red) (EEP exposed). The red colouration of propidium iodide in both extracts indicates greater damage to the yeast cells (40× magnification).

Figure 4 shows the effects of both EEPs on the clinical strain and also shows the staining of the untreated yeasts with calcofluor white but not with propidium iodide, which indicates the integrity of the plasma membrane and cell wall. The yeast samples treated with the EEPs of *S. mexicana* and *T. angustula* exhibited a similar effect. In both cases, the yeast samples were not stained with calcofluor white but were stained with propidium iodide, which indicates that the EEP damaged the structure of the fungi. Considering both the reference strain and the clinical one, we can conclude that the EEPs of *S. mexicana* and *T. angustula* affect the structural integrity of the yeasts, and this is more evident in the clinical strain.

## 4. Discussion

Many of the identified metabolites of stingless bee propolis were reported to exhibit a myriad of different chemical compositions, which is consistent with other studies that have biological activities, including antimicrobial, antiinflammatory, cytotoxic, antioxidant, hepatoprotective, and antiulcer effects [41,42,43,44,45]. The propolis extracts analysed show a diversity of compounds [46,47]. The floral diversity, time of collection, and bee species are all determinant factors for the final composition of each propolis, where sesquiterpene compounds predominated; these compounds are known for their antimicrobial activity [1,3].

Regarding the propolis of *Scaptotrigona mexicana*, bibliographic research of furan-2,5-dicarbaldehyde (a heterocyclic compound with two aldehyde groups) was unsuccessful using that exact denomination; however, we found mention of a similar compound, 2-acetyl-5-methylfuran, which was reported to exhibit antimicrobial activity against *Escherichia coli*, *Candida albicans*, and *Staphylococcus aureus* [32,33,39].

On the other hand, as the propolis of *Tetragonisca angustula*, the antibacterial and antifungal activity of solavetivone has been reported [35,36]. There is no specific information on the activity of l-(1S,6R,9S)-5,5,9,10-tetramethyltricyclo[7.3.0.0(1,6)]dodec-10(11)-ene; however, antifungal and antibacterial activities have been reported for a similar compound, 3,3,7,7-tetramethyl-5-(2-methyl-1-propenyl)-tricyclo[4.1.0.0(2,4)]heptane [38].

A notable difference was observed between the chemical composition of the analysed propolis and that of Mexican *Apis mellifera.* The antimicrobial activity of *Apis mellifera* propolis is related to the presence of flavonoids such as pinocembrin, tectochrysin (flavone), and the flavonoid precursor cardamomin (chalcone), as well as 2-methoxy-4-vinylphenol and a few terpenoids [10]. On the other hand, in this work, sesquiterpenes were the most important compounds, which is in agreement with a study by Bankova [2], which showed that terpenoids predominate in the propolis of native bees from various parts of the world. This seems to be a marked difference between the propolis of honeybees and native bees.

The antimycotic activity of propolis extracts, mainly from *Apis mellifera*, was demonstrated against *Candida albicans*. Moreover, the fungicidal and fungistatic properties of green and red propolis extracts from Brazil against other fungi genera were reported [43]. In addition, the inhibition and morphologic alterations of *Cryptococcus neoformans* when exposed to propolis were described [46].

The antimycotic activity of propolis from stingless bees, mainly against *Candida albicans*, has been reported for the following species: *Lestrimellata* spp., *Melipona favora orlinge*, *Melipona marginata*, *Melipona quadrifasciata*, *Melipona scutellaris*, *Nannotrigona testaceicornis*, *Plebeia droryana*, *Plebeia remota*, *Scaptotrigona bipunctata*, *Tetragona clavipes*, *Tetragonisca angustula*, and *Tetragonisca fiebrigi* (against *Candida glabrata*) [2,47]. The propolis from the Malaysian stingless bee *Trigona thoracica* was demonstrated to act against *Cryptococcus neoformans* [47]. Furthermore, an Indonesian propolis from *Tetragonula* sp. was evaluated as a possible therapeutic agent for the treatment of vaginal candidiasis [48]. However, no studies on the use of the propolis from this stingless bee for antifungal applications in animals were found.

Some reports have evaluated the activity of the propolis from *Apis mellifera* against *Malassezia pachydermatis*, but there are no such reports focusing on native bees. In a recent study, a correlation was established between the antimycotic activity of the ethanolic extract of Brazilian green and red propolis against *M. pachydermatis*, with an MIC between 4 and 8 mg/mL and an MFC of 8–16 mg/mL [49]. The study reported that, as the total content of phenols and flavonoids increased, propolis exhibited an enhanced biological effect, suggesting that the mechanism of action of EEP is based on the rupture of the cell wall. This idea is reinforced by the observation that some azole-resistant *M. pachydermatis* strains were inhibited by the EEP. The efficacy of an Argentinian propolis against *M. pachydermatis* was evaluated by different in vitro techniques; the results demonstrated that the yeast was vulnerable to all tested propolis concentrations, with an MIC of 0.30 mg/mL; however, the researchers were unable to determine the MFC [17]. The efficacy of a 2.5% EEP solution against 48 clinical strains of *M. pachydermatis* isolated from dogs diagnosed with otitis externa was also proven, as it was found that all the strains were susceptible to the EEP solution [17]. An EEP from Rio Grande do Soul, Brazil, demonstrated antimycotic activity against clinical isolates from dogs with otitis externa, with an MIC of 2.6 mg/mL and an MFC of 5.3 mg/mL. In this work, an MFC of 21.3 mg/mL was determined, being higher than what was found with *Apis mellifera* propolis, but the MIC of 7.11 mg/mL determined was similar to what was reported for this propolis; however, it is unclear whether high EPP concentrations could induce cytotoxicity. Therefore, more research is needed to identify the active principles of propolis as well as their action mechanisms. Currently, there are two theories aiming to explain the antifungal activity of propolis: the first proposes that propolis elicits cellular wall lysis. and the other proposes that propolis damages the plasma membrane by inhibiting ergosterol synthesis [16].

The photomicrographs obtained in the present report revealed that the EEP was able to penetrate the plasma membrane, which was found using the minimum fungicidal concentration (21.33 mg/mL), causing severe damage and eventually the death of yeast samples through structural and functional damage caused by membrane disruption. Calcofluor white exhibits a high affinity for fungal wall components [50,51]. The alterations observed with this stain were mainly deformed morphologies. In some cases, we hypothesise that the complete destruction of the cell wall prevented the observation of the yeasts, which would be a possible effect of the sesquiterpenes present in the EEP, as previously described [52]. It would be advisable to perform computational chemistry studies to establish the extent of the damage to the yeast’s cell wall caused by these compounds.

On the other hand, propidium iodide binds to nucleic acids and increases red colouration when there is damage to the cell membranes. This red colouration indicates severe cell damage and death, which was observed in the reference strain and the clinical strains treated with both EEPs, demonstrating the effectiveness of this type of propolis. This effect with propidium iodide has been observed in other fungi, such as *Fusarium*, by evaluating naturally occurring compounds’ efficacy against fungal growth [19]. It is likely that using higher concentrations of EPP would have resulted in more damage being detected, so it would be advisable to use higher concentrations in future research.

The above-mentioned stains have been used to detect cellular damage by *Apis mellifera* propolis in yeasts of medical importance, such as *Candida albicans* [53,54,55] as well as the bacterium *Staphylococcus aureus* [56]. There is scant research on the antimicrobial activity of stingless bee propolis against infectious agents in animals, which represents an opportunity to improve animal health.

Our team demonstrated the antiviral activity of the propolis of the stingless bee *Plebeia frontalis* against the canine distemper virus. This encourages further investigation into its effects on other viruses that affect animals [57].

Therefore, this work demonstrates that the propolis of the Mexican stingless bees *Scaptotrigona mexicana* and *Tetragonisca angustula* has antimycotic effects and causes structural damage to *Malassezia pachydermatis*. This result supports the use of these types of propolis for therapeutic purposes. The findings of this study and their implications should be discussed in the broadest possible context. Future research directions may also be highlighted.

## 5. Conclusions

In conclusion, the present study has shown the antifungal properties and extent of structural damage caused by two propolis ethanolic extracts from two stingless bee species (*Scaptotrigona mexicana* and *Tetragonisca angustula)* found in the Mexican municipalities of Yecuatla, Veracruz, and Chalchihuitan, Chiapas, against different *Malazessia pachydermatis* strains, one as a reference (ATCC 14522) and three clinical isolates. Sesquiterpenes, along with other compounds, are possibly the reason for the antimycotic activity of both extracts. It is important to mention that, to our knowledge, the present work is the first to demonstrate the structural damage caused by and antifungal effects of Mexican stingless bee propolis against *Malazessia pachydermatis*.

Nonetheless, further research must be undertaken in order to provide a more solid scientific basis for the future employment of propolis as an alternative treatment for canine external otitis.

## Figures and Tables

**Table 1:** Constituents of Mexican *Scaptotrigona mexicana* propolis characterised by CG-MS.

Retention Time (min)	Compound Proposed by the Database	Chemical Classification	Biological Activity	Reference
30.60	(1.alpha.,4.alpha.,4a.alpha.,10a.alpha)-1,4,4a,5,6,9,10,10a-octahydro-11,11-dimethyl-1,4-methanocycloocta[d]pyridazine	Pyridazine (heterocyclic compound)	Antioxidant	[28]
31.58	Farnesol Isomer a	Sesquiterpene	Antimicrobial	[29]
32.48	Ethanone,1-(1,3a,4,5,6,7-hexahydro-4-hydroxy-3,8-dimethyl-5-azulenyl)-	Sesquiterpene ketone	Antimicrobial	[30]
33.68	3,4,5,6,7,8,9,10,11,12,13,14-Dodecahydro-18,18a- benzoxacyclohexadecin-16(18aH)-one dihydroxy methyl-2*H*-1--2-	Macrocycle	Activity not reported	[31]
33.73	Furan-2,5-dicarbaldehyde	Heterocyclic compound with aldehyde groups	Antioxidant, antimicrobial	[32,33]

Main compounds identified with an accurate identification (>90%) as related to the equipment database.

**Table 2:** Main constituents of propolis of *Tetragonisca angustula* characterised by CG-MS.

Retention Time (min)	Compound Proposed by the Database	Chemical Classification	Biological Activity	Reference
23.17	Hexadecanoic acid ethyl ester	Fatty acid	Antioxidant, hypocholesterolemic, nematicide pesticide	[34]
25.20	9-Octadecenoic acid, ethyl ester	Fatty acid	Antiinflammatory	[35]
28.98	1-(1,1-dimethylethoxy)-4-methylbenzene	Impurity	Not founded information	
30.68	Solavetivone	Sesquiterpenoid and a cyclic ketone	Antifungal, antiinflammatory	[36,37]
30.90	Benzene, 1-(1,1-dimethylpropoxy)-4-methyl	Impurity	No information found	
31.65	2-Methyl-3-(3-methyl-but-2-enyl)-2-(4-methyl-pent-3-enyl)-oxethane	Heterocycle Compound derivative	No information found	
32.75	(1S,6R,9S)-5,5,9,10-Tetramethyltricycle[7.3.0.0(1,6)]dodec-10(11)-ene	Sesquiterpene	Antibacterial antifungal	[38,39]
33.75	1,3-Benzenediol, 5-hexyl	Resorcinol derivative	Antibacterial, anthelmintic, local anaesthetic	[40]

Main compounds identified with an accurate identification (>90%) as related to the equipment database.

**Table 3:** Minimum inhibitory concentration (MIC) and minimum fungicidal concentration (MFC) values of stingless bee propolis extracts from two regions of the Mexican Republic on the reference strain *M. pachydermatis* ATCC 14522 and strains isolated from clinical samples.

Mexican Stingless Bee Species	Origin	*M. pachydermatis* ATCC 14522		Isolation Clinical *		Number of Isolates Clinical Inhibited *
		MIC (mg/mL)	MFC (mg/mL)	Media MIC (mg/mL)	Media MFC (mg/mL)	
*Scaptotrigona* *mexicana*	Yecuatla, Veracruz	7.11	21.33	7.11	21.33	3
*Tetragonisca* *angustula*	Chalchihuitan Chiapas	7.11	21.33	7.11	21.33	3

* *n* = 3.

## Data Availability

Data are contained within the article.
